# The natural history of latent rheumatic heart disease in a 5 year follow-up study: a prospective observational study

**DOI:** 10.1186/s12872-016-0225-3

**Published:** 2016-02-19

**Authors:** Liesl Zühlke, Mark E. Engel, Carolina E. Lemmer, Marnie van de Wall, Simpiwe Nkepu, Alet Meiring, Michael Bestawros, Bongani M. Mayosi

**Affiliations:** The Cardiac Clinic, Department of Medicine, Groote Schuur Hospital and University of Cape Town, Cape Town, South Africa; Division of Paediatric Cardiology, Department of Paediatrics and Child Health, Red Cross War Memorial Children’s Hospital and University of Cape Town, Cape Town, South Africa; Present address: Division of Cardiology, Department of Internal Medicine, Tygerberg Hospital and Stellenbosch University, Parow, South Africa; Present address: New Mexico Heart Institute and the University of New Mexico, Albuquerque, NM USA

**Keywords:** Latent rheumatic heart disease, Natural history, Outcome

## Abstract

**Background:**

Latent rheumatic heart disease (RHD) occurs in asymptomatic individuals with echocardiographic evidence of RHD and no history of acute rheumatic fever. The natural history of latent RHD is unclear but has important clinical and economic implications about whether these children should receive penicillin prophylaxis or not. We performed a 5-year prospective study of this question.

**Methods:**

In August 2013 through September 2014, we conducted a follow-up study of latent RHD among school pupils using the World Heart Federation (WHF) echocardiographic criteria. Contingency tables were used to assess progression, persistence or regression of latent RHD.

**Results:**

Forty two borderline and 13 definite cases of RHD (n 55) were identified, 44 (80 %; mean age 13.8 ± 4.0 years; 29 (65.9 %) female) of whom were available for echocardiographic examination at a median follow-up of 60.8 months (interquartile range 51.3-63.5). Over the follow-up period, half the participants (*n* = 23; 52.3 %) improved to normal or better WHF category (regressors), a third (*n* = 14, 31.8 %) remained in the same category (persistors), while seven others (15.9 %) progressed from borderline to definite RHD (progressors). In total, 21 subjects (47.7 %) reverted to a normal status, nine (20.4 %) either improved from definite to borderline or remained in the borderline category, and 14 (31.8 %) either remained definite or progressed from borderline to a definite status. Two cases (20 %) progressed to symptomatic disease.

**Conclusions:**

Latent RHD has a variable natural history that ranges from regression to normal in nearly half of cases, to persistence, progression or development of symptoms in the remainder of subjects.

## Background

There is a heavy burden of rheumatic heart disease (RHD) in many developing countries and in some indigenous communities of developed countries [1, 2]. RHD exacts the highest number of disability-adjusted life-years of all cardiovascular diseases among 10–14-year-olds (516 · 6 per 100 000 people, 95 % CI 425 · 3–647 · 0) and the second highest number among children aged 5–9 years (362 · 0 per 100 000 people, 294 · 6–462 · 0) [3]. There is increasing recognition of the entity of latent RHD, which refers to individuals with echocardiographic evidence of RHD who have no history of acute rheumatic fever (ARF) and no symptoms. [4, 5] It is possible that the early institution of penicillin in individuals with latent RHD may prevent progression to overt clinical disease, but this has not been established in prospective studies [6, 7].

There are several limitations with the existing studies of the natural history of latent RHD detected by screening echocardiography [8–11]. First, three of the four studies of this question to date used non-standardised criteria for the diagnosis of RHD [8–10]. These criteria are associated with widely varying estimates of the prevalence of the disease even in the same population [12]. The World Heart Federation (WHF) has developed new criteria for the echocardiographic diagnosis of latent RHD that serve as the new consensus-based standard for research in this field and allows for comparison of data across communities and countries [13, 14]. These criteria make a distinction based on age, with no borderline category in those older than 20 years. Second, many of these studies have reported variable use of penicillin prophylaxis in patients with latent RHD, which may change the natural history of the condition. Third, not all patients with latent RHD were reported at follow-up, with some studies only reporting changes in participants with probable and possible disease [8]. Fourth, although two studies reported the follow-up of latent RHD by WHF criteria, they had relatively short follow-up periods of 27–30 months [11, 15]. Finally, there is no information on the outcome of latent RHD in subjects who are older than 18 years [16–18].

We have used the WHF criteria to assess the natural history of latent RHD disease diagnosed in childhood over a period of five years into late adolescence and early adulthood.

## Methods

### Study design

This is a prospective study of the natural history of latent RHD diagnosed by echocardiography during the course of a surveillance study in school pupils living in the residential areas of Bonteheuwel and Langa of Cape Town. The study protocol conforms to the ethical guidelines of the 1975 Declaration of Helsinki as reflected in a priori approval by the human research committee of the University of Cape Town. All participants gave written parental consent and individual assent prior to the enrolment echocardiogram.

The sampling frame, selection of participants, and echocardiographic protocol of the echocardiographic surveillance study in Cape Town have been described in full elsewhere [19]. The period of follow-up began in January 2008 with the enrolment of the first participant to September 2014 when the last participant was followed-up. The scoring of the enrolment and follow-up echocardiograms according to the WHF criteria occurred in August 2013 through September 2014.

We invited all those diagnosed with latent RHD according to the WHF criteria for a follow-up echocardiogram by one examiner (LZ). A second reader (AM, CEL, MB, or MvdW) reviewed the enrolment and follow-up echocardiograms before final classification into definite RHD, borderline RHD or normal study. The overall agreement for definite disease was good (kappa 0.77, 95 % confidence interval (CI) 0.71-0.83) and for borderline disease fair (kappa 0.69; 95 % CI 0.65 to 0.73). All readers used the same set of training echocardiograms to ensure consistent application of the WHF criteria.

### Variables and Measurement

The WHF criteria have two abnormal categories for those under the age of 20 years (i.e., borderline and definite disease) and one abnormal category for those over the age of 20 years (i.e., definite disease). We classified all our participants at follow-up according to those guidelines, with the age-specific cut-offs for morphological features [13]. Subsequent to this, we classified participants into the following categories based on the review of the enrolment and follow-up echocardiograms: persistors (i.e., diagnostic category unchanged), progressors (i.e., worsened, e.g., from borderline to definite) or regressors (i.e., improved from definite to borderline or from borderline/definite to normal).

### Statistical considerations

We used mean, median, standard deviation and interquartile range, where appropriate to describe the variables. We explored univariate relationships using cross-tabulations and calculated frequencies. We used contingency table analyses to test differences between proportions using Pearson-s Chi-squared test or Fisher’s exact tests as appropriate. This was followed by logistic regression analysis to identify risk factors for persistence or progression of RHD. We defined statistical significance as a two-sided *P* < 0.05. Epi-Info software was used to manage the data, and the statistical analysis was performed with Stata Version 11.2 (Cary, NC, USA).

## Results

### Participants

We invited 42 scholars with borderline and 13 with definite RHD at screening echocardiography for the follow-up examination about 5 years later (n 55, median age 13 years). Forty-four participants (80 %) with a median age of 18 years attended the follow-up echocardiogram. We could not contact eleven participants for the following reasons: nine (16 %) had left school with no contact details, one (2 %) was overseas and another (2 %) refused to return for follow-up. Table 1 shows the age, gender and follow-up duration of the 44 participants who underwent repeat echocardiography, two of whom (4.6 %) were on secondary antibiotic prophylaxis.

**Table 1:** Characteristics of the 44 schoolchildren with definite and probable rheumatic heart disease by the World Heart Federation criteria at follow-up

*Characteristic*	*Parameter* * (N =44)*		
Median age in years (IQR) (minimum, maximum) at initial diagnosis	13 (11–17) (5, 21)		
Female sex – *n* (%)	29 (65.9)		
Duration of follow-up, median months (IQR)	60.2 (51.5-63.5)		
Median age in years (IQR) (minimum, maximum) at follow-up	18 (17–22) (10, 26)		
*Changes in diagnosis of RHD by age at review visit*	*Under 20 years*	*Over 20 years*	*Total*
Regressors (improved) *n* (%)	12 (42.9)	11 (68.8)	23 (52.3)
Persistors (remained the same) *n* (%)	12 (42.9)	2 (12.5)	14 (31.8)
Progressors (lesions worsened) *n* (%)	4 (14.2)	3 (18.7)	7 (15.9)
Ratio of persistors and progressors to regressors	1.5	0.4	0.9

*IQR* interquartile range

### Natural history

**Fig. 1:**
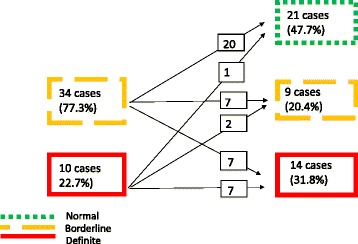
Natural history of latent rheumatic heart disease in school pupils after 60 months of follow-up

Half the participants (*n* = 23; 52.3 %) improved over the follow-up period to either borderline RHD or to a normal status. A third of the participants (*n* = 14, 31.8 %) remained in the same WHF category while seven others (15.9 %) worsened, progressing from borderline to definite RHD. The number of definite cases of RHD increased from 10 cases at baseline to 14 cases at follow-up (40 % increase). The number of borderline cases of RHD fell from 34 to nine while 21 became normal (47.7 %) (Fig. 1).

There were three types of outcomes of borderline RHD (n 34, 77.3 %): (a) regression to normal (n 20, 58.8 %), (b) persistent borderline state (n 7, 20.6 %), and (c) progression to definite RHD (n 7, 20.6 %) (Fig. 1). The 9 borderline cases that were identified at the follow-up visit had two patterns of valve involvement: either pathological mitral regurgitation (MR) (6 cases, 66.7 %) or at least two morphological abnormalities of the mitral valve (3 cases, 33.3 %). There were no cases of pathological aortic regurgitation (AR) or cases with two or more morphological features of the aortic valve.

Cases of definite RHD (n 10, 22.7 %) either remained unchanged (n 7, 70 %) or improved to a borderline status (n 2, 20 %) or reverted to normal (n 1, 10 %) (Fig. 1). The 14 cases of definite RHD that were identified at the follow-up visit displayed one of three patterns of valve disease: 12 cases (85.7 %) had pathological MR with at least two abnormal morphological features of the mitral valve; one case (7.1 %) had pathological AR with at least two abnormal features of the aortic valve; and one case (7.1 %) had borderline disease of both aortic and mitral valves. There were no cases of mitral stenosis.

There were 16 participants who were older than 20 years at follow-up (range 21–27 years); the WHF criteria classify borderline changes as normal in this age category. As a result, only five (31.3 %) still had evidence of latent RHD, while 11 (68.7 %) had reverted to normal. Of the five with latent RHD, two had persistent disease (12.5 %), whilst three had progressed from borderline to definite RHD (18.7 %).

### Factors to determine persistence or progression

There was no significant association of age at diagnosis (*P* = 0.582), gender (*P* = 0.61) or residential area (*P* = 0.08) with persistent or progressive RHD. On echocardiographic features, only pathological MR was associated with persistent RHD (*P* = 0.03). Pathological MR had an area under the receiver-operating characteristic curve of 0.69, 95 % CI 0.54 to 0.83 for predicting the persistence of either definite RHD or borderline RHD (Table 2).

**Table 2:** Echocardiographic features associated with progression or persistence of rheumatic valvular lesions

	*Normal on follow-up*	*RHD on follow-up*	*p*
	*N* = 21 (47.7.)	*N* = 23 (52.3)	
*Demographic features*			
Female gender	13 (44.8)	16 (55.2)	0.61
Langa suburb	17 (56.7)	13 (43.3)	0.08
*Echocardiographic features on screening*			
Pathological mitral regurgitation *n* (%)	9 (33.3)	18 (66.7)	0.03
Pathological mitral valve morphology *n* (%)	11 (55)	9 (45)	0.37
Anterior mitral valve thickness > = 3 mm	13 (44.8)	16 (55.2)	0.75
Chordal thickness	3 (33.3)	6 (66.7)	0.46
Restricted leaflet motion	10 (66.7)	5 (33.3)	0.11
Excessive leaflet motion	3 (60)	2 (40)	0.65
Pathological aortic regurgitation *n* (%)	2 (28.6)	5 (71.4)	0.42

### Symptomatic disease

There were two cases that developed clinical symptoms of RHD over the five year period. The first patient was a 16-year-old girl first seen four years and three months earlier with definite RHD (i.e., pathological MR and two additional morphological features of the mitral valve on echocardiogram). Upon follow-up, she presented with symptoms and signs of cardiac failure at eight weeks of pregnancy, and echocardiography revealed pathological MR and three abnormal morphological features of the mitral valve with normal left ventricular function. We commenced her on diuretics and referred her to a tertiary centre. She had been adherent to monthly benzathine penicillin and had no history of either sore throat or episodes of ARF.

The second patient was 22 years old, enrolled 5 years and 3 months prior to the follow-up visit with borderline RHD (pathological MR). She had tested positive for human immunodeficiency virus (HIV) prior to the follow-up visit but was non-adherent to anti-retroviral therapy. Upon follow-up, she had severe MR, an increased left ventricular end-diastolic diameter of 57 mm (Z score 2.55) and decreased left ventricular ejection fraction of 45 %. She had no history of sore throat or symptoms consistent with ARF and was subsequently lost to follow-up.

## Discussion

There are at least three key findings of this study. First, echocardiographic changes that were suggestive of latent RHD diagnosed by the WHF criteria in school pupils revert to normal in almost half the cases when followed for five years. This was more evident in those over the age of 20 years at follow-up, three quarters of whom reverted to normal. Second, definite or borderline RHD in asymptomatic scholars with no history of ARF is a dynamic phenotype, which may regress to normal, remain unchanged, or progress to more severe and even symptomatic disease in a small proportion affected individuals. Finally, the natural history of borderline RHD was associated with greater improvement than definite RHD, probably because of a lack of a borderline category in participants over the age of 20 years in the WHF criteria. More than half of the cases in the borderline category reverted to normal (*n* = 20, 58.8 %) compared to only one case (10 %) in the definite category. However, the two cases that progressed to symptomatic disease were in each diagnostic category, indicating that the borderline RHD category identifies cases that are at risk of clinical RHD.

**Fig. 2:**
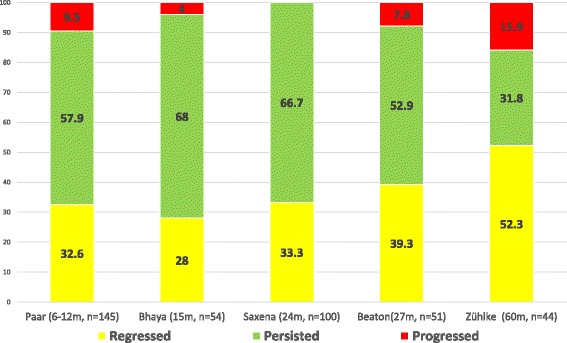
A comparison of the natural history of latent rheumatic heart disease in five studies with increasing durations of follow-up. Acronyms m = months of follow-up, *n* = sample size of follow-up cohort

This study extends the existing knowledge on the outcome of latent RHD in several ways. First, we show that the strict use of WHF criteria in subjects older than 20 years is associated with a greater proportion of cases that are classified as normal because of the lack of the category in this group. The clinical significance of the re-classification of participants with borderline changes to normal by virtue of age warrants further study. Second, we show that a longer duration of follow-up may be associated with a larger proportion of cases with improvement in rheumatic valvular lesions. Whereas previous studies with a follow-up ranging from 6 months to 30 months have shown improvement in 28 % to 39 % of cases [8–11], 52 % of cases improved in this study (Fig. 2). Finally, only 2/44 cases (4.6 %) were receiving secondary antibiotic prophylaxis during the follow-up period. This is therefore a true natural history study of latent RHD over a long period of time. It is of interest that one of the two patients on antibiotic prophylaxis in fact progressed to overt clinical RHD.

Previous natural history studies of latent RHD have reported the persistence or progression of lesions in 53-68 % of cases over 6 months to 30 months of follow-up; our study falls in the lower border of this estimate with about 48 % of persistence or progression (Fig. 2) [8–11]. Other studies have also found the predictive value of definite RHD and significant mitral valve disease for persistent or progressive disease. In contrast, other investigators have shown that morphologic abnormalities are associated with persistence and progression of disease, which was not the case in this study [11]. It is possible that the determination of morphological abnormality on the heart valves is subject to greater observer variation, confounded by technical settings of echocardiography, and affected by the prevalence of other conditions such as endomyocardial fibrosis in the background population.

Our study has several limitations. First, the sample size is small; it is therefore more likely to generate a hypothesis rather than provide a definite answer to the question of the natural history of echocardiographic RHD in asymptomatic school pupils. A prospective, international, multi-centre registry of definite and borderline RHD (known as the “DefineRHD” registry) is underway to address the concerns regarding the natural history in a large number of cases [5]. Second, we did not perform auscultation at the time of enrolment of the participants in this study. This decision was made on the basis of the superior performance of echocardiography in the detection of latent RHD [20]. Therefore this is a study of asymptomatic RHD, which cannot address the question of the outcome of subclinical (i.e., without murmur) versus clinical (i.e., with murmur) forms of latent RHD. Third, this study cannot make inferences regarding the time interval from the initial attack of ARF to entry into this five year observation period. It is possible that individuals in their third decade actually had an attack of ARF more than 10 years previously while younger individuals had the attack of ARF only one or two or even three years prior to being enrolled in the study. A recent paper [15] suggests that screened children in an endemic area should be followed as both groups (latent RHD and initially normal) can develop episodes of ARF in the following years. Finally, the 20 % loss to follow-up rate is a matter of concern, but is comparable to other studies in this field due to migration of participants after leaving school [8–11].

We undertook several measures to minimise bias in this study. Firstly, the random ascertainment of the participants in the surveillance sought to ensure the generalizability of finding to school going population of the Bonteheuwel and Langa communities of Cape Town. Secondly, we invited all available participants with abnormal enrolment echocardiograms to participate in the follow-up study. We based our study size therefore on the number of participants with abnormal echocardiograms identified at the time of original ascertainment. Finally, one observer applied the WHF criteria to the baseline and follow-up echocardiograms, with verification of findings by a second observer.

Despite the limitations, our findings do have important implications for clinical practice and research. It is clear from this and other studies that a diagnosis of latent RHD on echocardiography requires confirmation in follow-up studies due to the dynamic nature of the condition – which may resolve, persist or deteriorate [8–11]. According to existing studies, a minimum follow-up period of 12 months may be reasonable to identify cases who regress to normal, although our study suggests that a longer duration of up to five years (into late adolescence and early adulthood) may identify a higher proportion of cases that regress to normal. The management of those with persistent subclinical RHD is unknown [21]. Whilst some authorities have recommended the institution of secondary antibiotic prophylaxis to prevent progression to overt RHD, this advice is not based on randomised controlled evidence [21, 22]. Therefore, there is need for adequately powered randomised controlled trials of antibiotic prophylaxis to determine the effectiveness and safety of secondary antibiotic prophylaxis in preventing morbidity and mortality associated with persistent latent RHD [6, 21].

## Conclusions

Latent RHD resolves to normal in nearly half of school pupils followed up for 5 years. There is therefore a need to repeat echocardiography in these cases before an intervention is considered. The effectiveness and safety of secondary antibiotic prophylaxis in persistent latent RHD needs to be tested in large randomised controlled trials.

## Abbreviations

AR: Aortic regurgitation
ARF: Acute rheumatic fever
HIV: Human immunodeficiency virus
MR: Mitral regurgitation
RHD: Rheumatic heart disease
WHF: World Heart Federation
